# The electrochemical kinetics of cerium selenide nano-pebbles: the design of a device-grade symmetric configured wide-potential flexible solid-state supercapacitor†

**DOI:** 10.1039/d0na00893a

**Published:** 2020-12-21

**Authors:** Bidhan Pandit, Akanksha Agarwal, Priyanka Patel, Babasaheb R. Sankapal

**Affiliations:** Nano Materials and Device Laboratory, Department of Physics, Visvesvaraya National Institute of Technology South Ambazari Road Nagpur 440010 Maharashtra India brsankapal@phy.vnit.ac.in brsankapal@gmail.com +91 712 2223230 +91 712 2801170; Institut Charles Gerhardt Montpellier (ICGM), Université de Montpellier, CNRS Place Eugène Bataillon Montpellier 34095, Cedex 5 France

## Abstract

Next-generation portable flexible electronic appliances require liquid-free energy storage supercapacitor devices to eliminate leakage and to support mechanical bending that is compatible with roll-to-roll technologies. Hence, a state-of-the-art process is presented to design a solid-state, wide-potential and flexible supercapacitor through the use of nano-pebbles of cerium selenide *via* a simple successive ionic layer adsorption and reaction (SILAR) method that could allow an industry scalable route. We strongly believe that this is the first approach amongst physical and chemical routes not only for synthesizing cerium selenide in thin-film form but also using it for device-grade supercapacitor applications. The designed solid-state symmetric supercapacitor assembled from cerium selenide electrodes sandwiched by PVA–LiClO_4_ gel electrolyte attains a wide potential window of 1.8 V with capacitance of 48.8 F g^−1^ at 2 mV s^−1^ and reveals excellent power density of 4.89 kW kg^−1^ at an energy density of 11.63 W h kg^−1^. The formed device is capable of 87% capacitive retention even at a mechanical bending angle of 175°. Lighting up a strip of 21 parallel connected red LEDs clearly demonstrates the practical use of the designed symmetric solid-state supercapacitor, aiming towards the commercialization of the product in the future.

## Abstract 2

Chemically synthesized cerium selenide for high performance symmetric solid-state supercapacitors.

## Graphical abstract

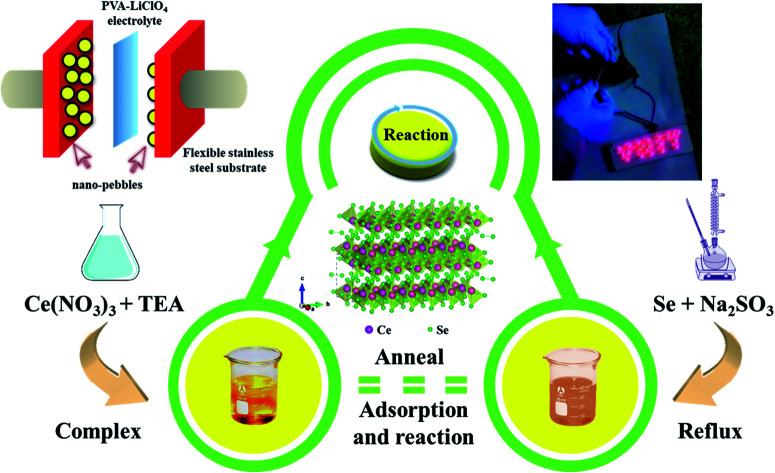

## 1. Introduction

The excellent electronic structure with outstanding physical properties of nanostructured materials has led to them attracting significant attention towards electrochemical applications compared to the bulk.^1^ Bulk material has limited utility in electrochemical charge storage applications due to slow rate of electrochemical redox reactions. Forming nanostructures can be a viable solution to overcome such limitations by accessing both surface and bulk redox sites.^2^ In the nano regime, electrochemical activities can dominantly be boosted from rapid diffusion path length for ion transportation and, hence, increases prospects for faradaic reactions on the surface.^3^

Cerium is well defined as a common rare-earth element, and it is the 25th most abundant element on the Earth with analogous abundance of copper.^4^ Interestingly, environment-friendly features of cerium and cerium-based chalcogenides make them even more important materials for widespread use in the context of future energy storage applications. Furthermore, Ce(iv) attracts much interest with electronic state configuration 4f^1^5d^1^6s^2^. Due to the toggling oxidation states of this element (*e.g.* Ce^3+^ ↔ Ce^4+^), it has been found to be energetically too demanding. Furthermore, cerium (Ce) is well known for nanoscale properties that can be utilized to improve charge storing rates for the same material which displays poor kinetics in normal bulk form. The porous framework and unique nanoscale periodicity of cerium lead to short diffusion paths with more active sites which help to improve charge storage capability. Due to its exceptional properties, cerium has attracted attention for wide-ranging applications such as gas sensing (oxygen), catalysis, and electrolyte in fuel cells (especially solid oxide).^5–7^ Oxides and chalcogenides of cerium as well as corresponding composites are synthesized to engineer the morphology and structure more efficiently for charge storage *via* numerous methods including template-assisted and hydrothermal synthesis where capacitance ranges from a few hundred up to greater than a thousand farads.

Selenium is the closest neighboring element of sulfur in VI-A group and it has similar oxidation number and valence electrons to sulfur.^8^ The electrochemical kinetics including chemical behaviors of metal selenides resemble those of metal sulfides, indicating the promising applicability of metal selenides in supercapacitors (SCs).^9^ By analyzing nanocrystalline selenides, the energy kinetics might be significantly reduced due an increase in the non-stoichiometric levels, the defect level and the generation of electronic carriers.^10^ The van der Waals force is weaker at the selenium metal layer and facilitates the introduction of host material. In MSe_2_ structure, the metal atom is compacted among two selenium atoms through covalent bonds, signifying strong Se–M–Se association.^11,12^

Recently, transition metal selenides have shown excellent energy storage properties.^13,14^ Interconnected CoSe ultrathin nanosheets with 3D structure synthesized with a hydrothermal technique^15^ showed maximum capacity of 70.6 mA h g^−1^ (at current rate of 1 A g^−1^) with noteworthy 52.8% capacity retention for even 100 A g^−1^ current rate. A CoSe_2_ electrode prepared by an anion exchange reaction under a hydrothermal process attained maximum capacitance of 951 F g^−1^ at constant 5 mV s^−1^ scan rate with decent capacitive retention for 2500 cycles.^16^ NiSe nanowires were formed on a nickel foam surface by using a hydrothermal method to afford a good capacitance value of 1790 F g^−1^ with specific 5 A g^−1^ current density, as reported by Tang *et al.*^17^ Pseudocapacitive binder-free CuSe_2_/Cu was prepared by easy hydrothermal condition, and the associated electrode delivered excellent capacitance of 1037.5 F g^−1^ at fixed 0.25 mA cm^−2^ current rate.^10^ (Ni, Co)_0.85_Se reported by Xia *et al.* was capable of exhibiting a superior areal capacitance of 2.33 F cm^−2^ for a current rate at 4 mA cm^−2^.^18^ Comparably, an electrode prepared with Ni–Co–Se nanowire morphology revealed a maximum capacitance of 86 F g^−1^ for current rate of 1 A g^−1^ and outstanding cyclic stability, through nearly no diminution in capacitance after 2000 charge–discharge cycles.^19^ Thus, transition metal selenides with excellent electrochemical energy storage capacities have been upgraded compared to other electrode materials studied for SC applications. To date, no attempt has been made to prepare cerium selenide *via* physical or chemical routes and to apply it as an electrode for energy storage applications.

The present effort is focused on the synthesis of nano-pebbles of cerium selenide to develop a supercapacitive electrode to strategize a full solid-state and bendable SC. With the aid of a low-cost, simple, and industry mountable chemical route, a successive ionic layer adsorption and reaction (SILAR) method has been established to develop a device grade SC at lower cost having greater stability and specific capacitance. A literature survey supports well the capability of porous metal selenide film allowing enhanced interaction with electrolytes at the atomic level due to weak van der Waals force. Furthermore, the oxidation state of cerium switches among Ce^3+^ and Ce^4+^ due to toggling behavior owing to its electronic configuration yielding more capability to store more charges through the faradic reversible redox reactions. Hence, cerium has been selected with selenium to form a cerium selenide electrode to develop a pseudocapacitor. It has been subjected to various electrochemical characterizations to support its energy storing capability and demonstration of a working model to manifest its practical applicability by lighting a VNIT logo involving 21 red light-emitting diodes (LEDs).

## 2. Experimental parameters

### 2.1 Electrode preparation and device assembly

Conducting stainless steel (SS) as a substrate to deposit cerium selenide nanostructured thin film offers many advantages such as lightweight, low cost and good mechanical strength towards flexibility and, importantly, widespread availability. Proper cleaning of the substrate plays a crucial role in film deposition to eliminate surface contamination which competes for nucleation sites resulting in non-uniform film formation. Hence, the SS substrate was cleaned properly through the following steps: initial cleaning using zero grade polish paper, rinsing in doubled distilled water (DDW), ultra-sonication in DDW for 15 min, finally rinsing in DDW again and drying completely in a vacuum oven at 80 °C overnight.

The SILAR approach involves immersion of the substrate into individually prepared cationic and anionic precursors with additional immersion step in DDW to remove unreacted species resulting in homogeneous film growth. Cationic precursor of 20 mM of cerium nitrate ((CeNO_3_)_3_) was prepared with addition of a few drops of triethanolamine (TEA, C_6_H_15_NO_3_). An anionic precursor of sodium selenosulfate (Na_2_SeSO_3_) was prepared by using a reflux process using 5 g of selenium (Se) and 15 g of sodium sulfite (Na_2_SO_3_) with 200 ml of DDW at 90 °C for 9 h followed by filtration. Initially, the SS substrate was immersed in cationic precursor for 50 s, where TEA-complexed cations were adsorbed on the substrate surface.^20^ The purpose of rinsing in DDW after adsorption is to remove the loosely adsorbed cations from the substrate surface. Next step involved the immersion of the substrate into anionic precursor for 50 s resulting in brown-colored deposition of intermediate cerium selenide. Completion of the cycle involved rinsing of substrate to remove excess and unreacted ions, and by-products derived from the diffusion layer. This sequence was repeated 40 times followed by annealing at 300 °C for 3 h to acquire higher oxidized and well-structured cerium selenide film on SS substrate with excellent adhesive properties (Fig. S1†).

The mechanism of cerium selenide deposition through SILAR involves the migration near the substrate surface for immediate adsorption after SS immersion. The associated metal ions are present in the form of isolated ions rather than solvated ions, which is why there is improvement in migration rate of ions that directly helps the growth after nucleation. Subsequently, TEA-complexed cerium ions are prone to react with slow release of Se^2−^ ions from HSe^−^ as intermediate during the reaction process yielding the formation of cerium selenide to grow on the SS substrate:^21,22^

1 Na_2_SeSO_3_ + OH^−^ → Na_2_SO_4_ + HSe^−^

2 HSe^−^ + OH^−^ → Se^2−^ + H_2_O

**Fig. 1:**
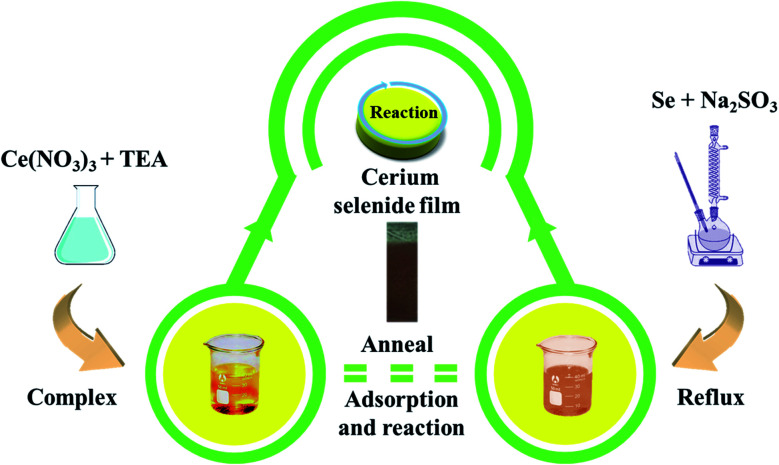
A schematic diagram of cerium selenide synthesis using a chemical route.

Finally, the important annealing process helps to rid the cerium selenium complex of any inclusion of hydroxyl content, affording well-structured cerium selenide on the SS substrate surface. A complete schematic for the process is visualized in Fig. 1.

### 2.2 Characterization

For crystallographic information, structure, and composition of electrode materials, the X-ray diffraction (XRD, Bruker AXS D8) and X-ray photoelectron spectroscopy (XPS, PHI 5000 VersaProbe II ULVAC INC) techniques were used. Belsorb apparatus was employed to calculate the surface area (Brunauer–Emmett–Teller (BET) equation) and pore-size distributions (Barrett–Joyner–Halenda (BJH) method) of the sample outgassed in a vacuum at 200 °C for 12 h before analysis. Field emission scanning electron microscopy (FESEM, JEOL-JSM 6360) and high-resolution transmission electron microscopy (HRTEM, JEOL 2100) were used to confirm the nanostructures and the morphology evolution of formed material. The capacitance performance of constructed electrodes was tested in a 3-electrode electrochemical cell setup with a PARSTAT-4000 potentiostat/galvanostat (Princeton Applied Research, USA) electrochemical workstation. The fabricated electrode with dimensions of 1 × 1 cm^2^ acted as working electrode with Ag/AgCl and platinum wire serving as the reference and counter electrodes, respectively, for electrochemical analysis.

Quasi-flexible solid-state symmetric supercapacitor (SSC) was constructed by involving two similar cerium selenide films obviously using PVA–LiClO_4_ polymer gel electrolyte in a sandwich structure. Unique PVA–LiClO_4_ gel electrolyte was synthesized by adding PVA (1 g) and LiClO_4_ (1 g) in 10 ml of DDW and maintaining at 90 °C for 12 h under vigorous stirring.^23–25^ Afterwards, two similar one-sided films were completely dipped in the prepared gel electrolyte for almost 10 min and after kept in open atmosphere for drying process. When the polymer gel became semi-solidified, two as-prepared films were placed in a sandwich-like structure and packed up to prepare a flexible symmetric supercapacitor (FS-SC) cell^26^ by applying 1 ton pressure normal to the plane of the substrate overnight ensuring complete drying of the semi-solidified gel to form a complete solid-state device along with better adhesion of the two sandwiched electrodes.

## 3. Results and discussion

### 3.1 Structural analysis

**Fig. 2:**
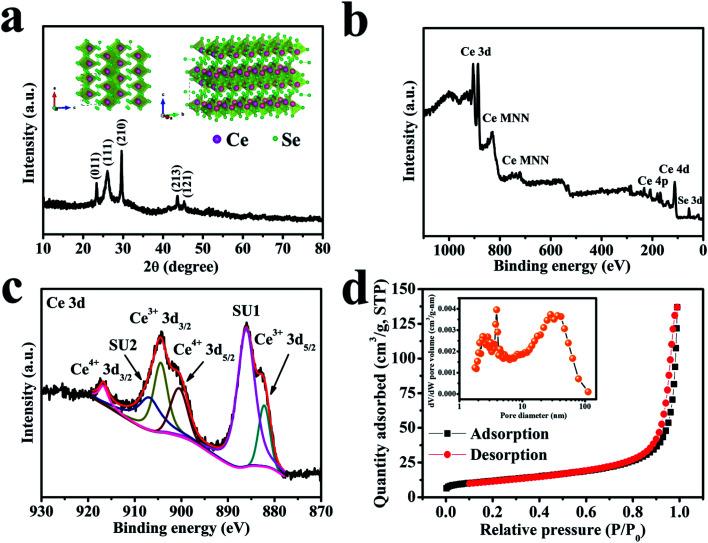
(a) The XRD pattern of a cerium selenide powder sample. The inset shows the corresponding crystal structure. (b) The survey XPS spectrum of a cerium selenide sample. (c) The core-level XPS spectrum of Ce 3d. (d) The nitrogen adsorption/desorption isotherm curve of cerium selenide. The inset shows the BJH pore size distribution plot.

The structural property of the cerium selenide powder sample was examined by XRD as depicted in Fig. 2a. Three clear intense peaks at 2*θ* values of 23.5, 25.9, 29.9° corresponding to (011), (111), (210) planes and two more moderate peaks at 2*θ* values of 44.0 and 45.6° which correspond to (213) and (121) planes indicate monoclinic cerium selenide (JCPDS card no. 74-1279) with a space group of P21/a, whose crystal structure is displayed in the inset of Fig. 2a. The typical open framework structure with Se-coordinated Ce cations is efficient for allowing electrolyte ions to participate in electrochemical activities.

The valence state of deposited material was examined by XPS analysis and the results are shown in Fig. 2b which is analogous to the literature^27,28^ where MNN is the Auger group of Ce.^29^ The maximum content of the Ce 3d core can be established by XPS investigation of the sample (see Fig. 2c) where the leading peaks of Ce^4+^ 3d_3/2_ and Ce^4+^ 3d_5/2_ appeared at binding energies of 916.7 and 899.1 eV, respectively. Weaker peaks of Ce^3+^ 3d_3/2_ and Ce^3+^ 3d_5/2_ appeared at 904.4 and 882.2 eV, respectively, due to the oxygen defects on the sample surface.^30^ Two similar satellite peaks SU1 and SU2 appear at 906.8 and 886.1 eV, respectively. The Ce 3d spectrum corresponds to that of an earlier report.^31^ The occurrence of trivalent Ce^3+^ may be due to surface defects.^32^ The conversion between Ce^4+^ and Ce^3+^ may be of crucial concern in electrochemical activities. It is evident that cerium selenide has an active release and storage capacitance through the redox shift among Ce^4+^ and Ce^3+^ regarding oxidation and reduction, respectively.

The surface area and pore-size distributions were calculated using the BET equation and BJH method, respectively. The BET analysis with nitrogen adsorption–desorption isotherms is shown in Fig. 2d. The cerium selenide nanostructure shows BET surface area of 41.5 m^2^ g^−1^ which assists in improving the supercapacitive performance of the electrode. The BJH pore size distribution analysis specifies that the as-prepared nano-pebbles have a pore diameter in the range of 3.9 to 36.3 nm (inset, Fig. 2d). The observed high surface area and pore volume facilitate the diffusion of electrolytes through the pores to access the maximum surface and reduce the electron transport path, beneficial during the electrochemical process in SCs.^33^

### 3.2 Morphological analysis

**Fig. 3:**
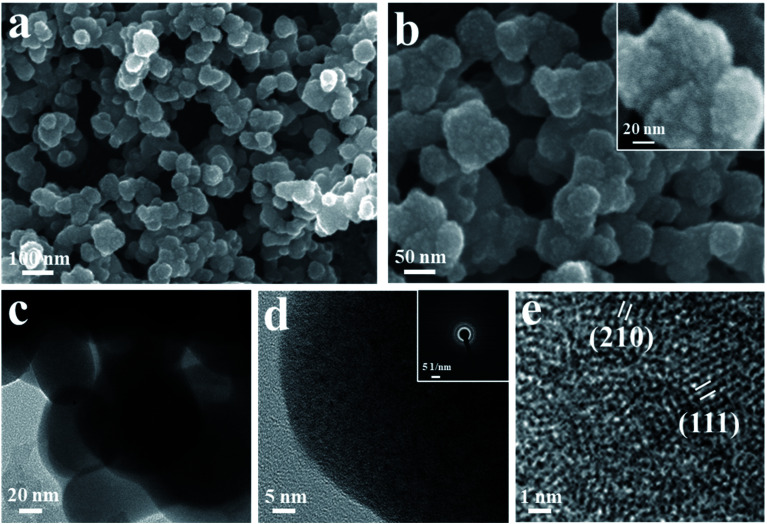
(a and b) FESEM images of cerium selenide thin film at various magnifications. The inset of (b) shows nanoparticle aggregation for nano-pebble formation. (c–e) HRTEM images of a cerium selenide sample. The inset of (d) shows the SAED pattern.

The FESEM images of cerium selenide film at different resolutions are depicted in Fig. 3a and b. The active electrode interface morphology of a material has a significant effect on electrochemical activity due to its presence over or close to the surface of the electrode. Fig. 3a shows the nano-pebble-like surface architecture of the synthesized cerium selenide. Fig. 3b shows the surface morphology of cerium selenide where the scale bar is 50 nm. After magnification, imaging gives a clear idea that the nano-pebbles consist of 5–6 nm sized nanoparticles. Here aggregation of nanoparticles yields a pebble-like surface architecture with high surface area which is a requisite for pseudocapacitive electrode material (inset, Fig. 3b). This surface structure provides a complex nano-network contributing to the charge storage on surface and inside volume as well, where the electrolytic ions have sufficient proximity to interact with the electrode material. All FESEM images illustrate that the complete substrate surface is uniformly organized by nano-pebbles embedded with nanoparticles.

The HRTEM images of electrode sample are depicted in Fig. 3c–e. Furthermore, the homocentric rings recorded in the SAED pattern also evidence the crystalline planes of the synthesized material (Fig. 3d, inset). The effect is seen due to the formation of bigger sized particles embedded with nanoparticles. Furthermore, the characteristic HRTEM image presented in Fig. 3e distinguishes interplanar spacings of lattice fringes as 0.34 and 0.29 nm, corresponding to (111) and (210) crystalline planes of cerium selenide, respectively.

### 3.3 Study of surface wettability

The water contact angle on the SS was determined to be 106°, which is greater than 90° and hence it shows a hydrophobic nature (Fig. S2†). Deposition of cerium selenide thin film on the SS substrate leads to a reduction of the contact angle from 106° to 33° exhibiting a hydrophilic nature responsible for high wettability and supports well the nano-porous surface architecture, useful for electrochemical studies of SCs.^34,35^

### 3.4 Supercapacitive performance of cerium selenide electrodes

Suitable choice of electrolyte and electrode is a crucial component to achieve wider potential window to gain higher charge storage capability and, hence, energy density. Organic non-aqueous electrolytes owning high decomposition voltage have been recognized to widen the electrochemical window which permits them to exhibit high energy density compared to aqueous electrolytes; however, their high-cost, flammable nature, lower ionic conductivity and swelling on repeated charge–discharge cycling limit their use.^36^ Furthermore, non-aqueous electrolytes always need an anhydrous environment to assemble devices, even though such trouble can be avoided using alternative aqueous electrolytes.^37^ In contrast, aqueous electrolytes are environmentally friendly and able to exhibit high ionic conductivity to show good power densities.^38^ The high proton mobility because of less weight, size, and related high ionic conductivity make aqueous electrolytes a good alternative option.^39^ Numerous research groups are working on environment-friendly and safe power sources for wearable and flexible electronic devices based on neutral salt electrolytes.^40^ Normally, a neutral salt electrolyte has lower concentration of H^+^ and OH^−^ delivering a wide potential frame without any gas evolution.^41^

**Fig. 4:**
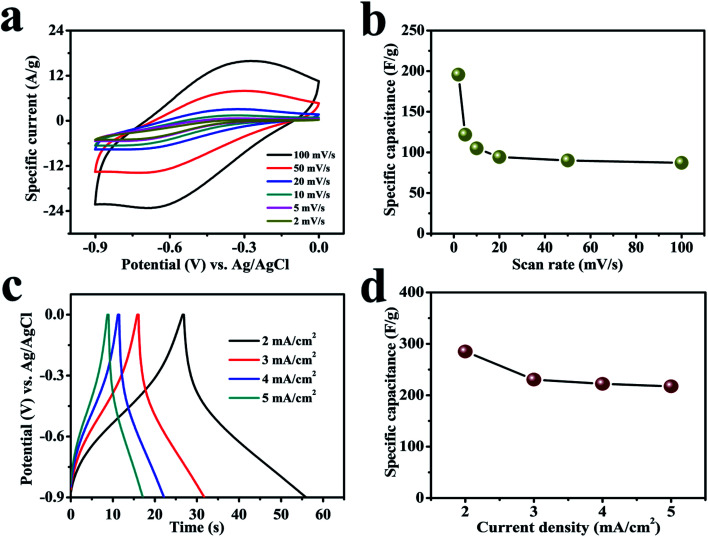
The electrochemical performance of the electrode in 1 M Na_2_SO_3_ electrolyte. (a) CV plots for a cerium selenide electrode at various scan rates. (b) Specific capacitance as a function of scan rate. (c) GCD curves at various current densities from 2 to 5 mA cm^−2^. (d) Specific capacitance as a function of current density.

Fig. 4a demonstrates digital CV curves with 100 mV s^−1^ scan rate that gradually reduced to 2 mV s^−1^ to compare the reversible redox reactions of a cerium selenide electrode in 1 M Na_2_SO_3_ aqueous electrolyte within a potential boundary of 0 to −0.9 V. The cerium selenide electrode deviates from rectangular shaped CV curve (EDLC behavior) by exhibiting clear oxidation and reduction peaks,^42,43^ which displays the reversible redox behavior of the cerium selenide electrode. The cerium selenide electrode exhibits a high current response owing to its unique nano-pebble-like surface architecture which enables more electrochemical pathways for electrolyte ions to penetrate. The equations used to evaluate the electrochemical parameters are specified in the ESI S3.† The comparatively higher capacitance of the electrode of 195.6 F g^−1^ at 2 mV s^−1^ (Fig. 4b) than at other scan rates is due to time constraint in the electrochemical process.^44^

A comparison of galvanostatic charge–discharge (GCD) plots was achieved with fixed current densities beginning with 2 mA cm^−2^ and then up to 5 mA cm^−2^ in the same potential window of 0 to −0.9 V (Fig. 4c). A small *IR* drop indicates a low internal impedance at the beginning of the discharge plot.^45^ The nonlinear GCD curves indicate the faradic reaction between as-prepared electrode and electrolyte.^46^ The electrode exhibits a superior specific capacitance of 285 F g^−1^ at constant current density of 2 mA cm^−2^ and offers the best permeable electrolyte ion channels. The contribution of the lower redox electroporation material shows a flux with higher current density coefficient; hence an increase in the current density leads to a decrease in the capacitance (Fig. 4d).

**Fig. 5:**
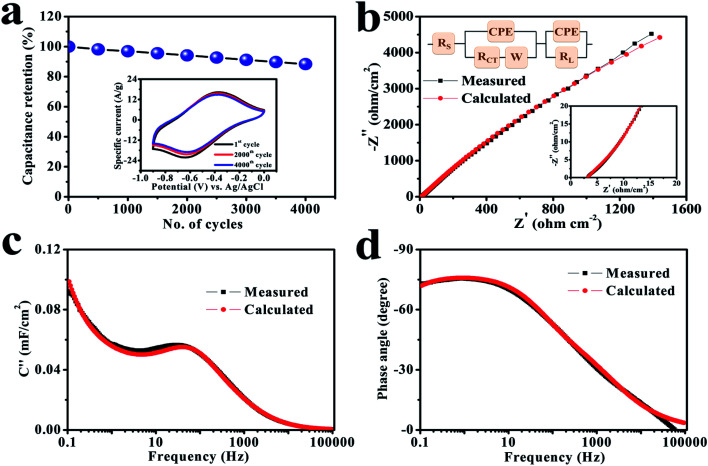
(a) Cycling stability of the electrode for 4000 cycles at a scan rate of 100 mV s^−1^. The inset shows CV plots after different numbers of cycles. (b) Nyquist plots of the electrode in the range of 100 kHz to 100 mHz. The insets display a magnified view of the high-frequency region and the associated equivalent circuit. (c) An imaginary capacitance *vs.* frequency plot and (d) the corresponding Bode plot.

Stability is an important factor for the actual application of prepared electrode materials, and hence a study was performed at a scan rate of 100 mV s^−1^ in equal potential limit where the involved electrode delivers an excellent retention by maintaining 88.4% of its primary capacitance over 4000 cycles (Fig. 5a), which reveals that the cerium selenide electrode not only has a good chemical stability in Na_2_SO_3_ electrolyte, but the time duration for power delivery also has a good reversibility.

The basis of the different electrochemical activities of electrodes can be evaluated more carefully from the EIS viewpoint, since it explains the internal capacitive and resistive processes of electroactive material. The EIS study was performed in a frequency range of 100 kHz to 100 mHz and results are depicted in Fig. 5b. The relatively lower frequency line specifies the net capacitive activities of the electrode in the Nyquist graph. In the high-frequency region (inset, Fig. 5b), the equivalent series resistance (*R*_S_) is represented in the first part of the *X*-axis, which is attributed to the resistance coefficients of the involved electrolyte.^47,48^ Alternatively, it also depends on the charge transfer resistance (*R*_CT_), to which the small semi-circular arc was applied in the higher frequency range; it is due to the morphology and structure of the electrode.^49,50^ In this situation, the electrode has low *R*_S_ (3.2 Ω cm^−2^) and *R*_CT_ (4.1 Ω cm^−2^) standards, which provide a good electrochemical reaction because of standard electrode–electrolyte interface. In fact, the conductive contact of the SS substrate and reduced interfacial charge transfer resistance improve the consumption rate of the electroactive material and, therefore, leads to an excellent capacity value. The result of the simulation combined with the equivalent circuit coincides with the experimental curve. The fitted parameters with chi-square statistics are listed in Table S1.† The constant phase element (CPE) is connected to electrolyte ion diffusion (semi-infinite), while the corresponding Warburg component (*W*) indicates the transition of spectrum of higher frequencies towards lower ones.^51,52^

The electrochemical performance rate can be well defined by the relaxation time constant (*τ*_0_), which evidently describes the limit among the ohmic and the capacitive activities.^53^ The small value of relaxation time constant (*τ*_0_) agrees with a higher conservation of power, favoring a supercapacitive nature. It can be analyzed by (*τ*_0_) = 1/*f*_0_ by considering the imaginary frequency component (*C*′′) *versus* frequency (*f*):^54–56^3
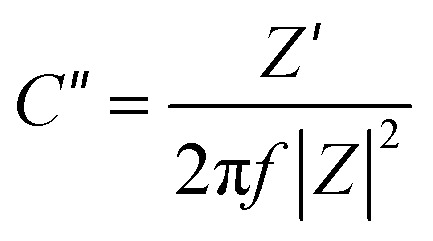


The electrode has a minimum value of relaxation time constant (*τ*_0_) of 29.8 ms (Fig. 5c), which obviously specifies the phenomenon of rapid charge transfer to internal pores through redox-active electrochemical activities.^57^ The Bode diagram differentiates the charging storage capacities of electrodes, having a dependency of a phase angle on the frequency. The comparative diagrams in Fig. 5d show that the high-frequency section acquires an analogous resistance. An ideal double-layer electrochemical capacitor has a familiar phase angle of 90°, while a pseudo-analyzer shows a change in phase angle.^58^ It shows a maximum phase angle of −75.5°, which indicates its suitability for the production of a leakage-free SC.^59^ The time constant is often an important quality factor for a SC as estimated by using the same equivalence *τ*_0_ = 1/*f*_0_ having a predicted angle phase of −45°, since resistive and capacitive impedances are similar.^60–63^ In addition, the rapid relaxation time (4.6 ms) of the electrode through the Bode design simplifies the quick diffusion of electrolyte ions even at the electrolyte–electrode interface.

### 3.5 Supercapacitive performance of the flexible solid-state symmetric device

**Fig. 6:**
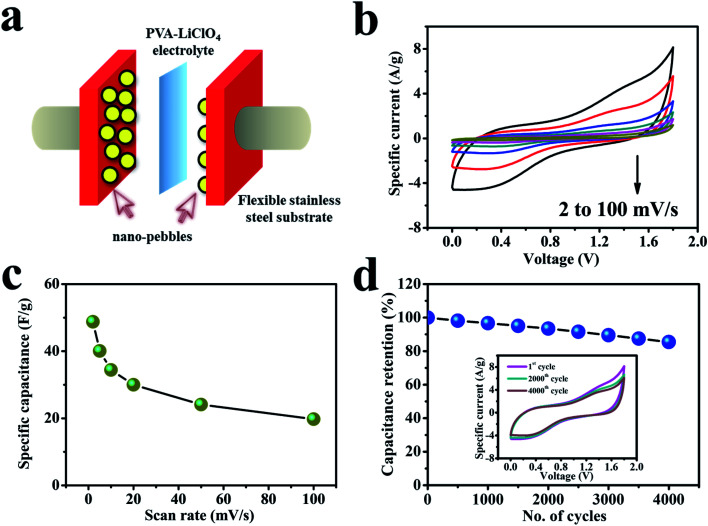
(a) A schematic diagram illustrating the solid-state device configuration. (b) CV plots of the symmetric solid-state device at different scan rates ranging from 100 to 2 mV s^−1^ with a voltage boundary of 1.8 V. (c) Specific capacitance as a function of scan rate. (d) Cycling stability of the device for 4000 cycles at a scan rate of 100 mV s^−1^. The inset shows CV plots after different numbers of cycles.

Energy storage devices have been enormously advanced matching with the extensive application of flexible devices. Therefore, cerium selenide based solid-state SCs were also constructed and explored in this work (named FS-SC). The fabrication of solid-state device is difficult with using PVA–Na_2_SO_3_ gel electrolyte, as it quickly solidified after preparation. The cause may be the non-compatibility of PVA with Na_2_SO_3_ electrolyte. In contrast, PVA–LiClO_4_ gel is highly conductive and, hence, most widely used during the fabrication of solid-state SC devices.^64–67^ Therefore, performance level of a solid-state device based on cerium selenide electrodes sandwiched by PVA–LiClO_4_ gel electrolyte^68^ was evaluated and a schematic illustration of the cell assembly is depicted in Fig. 6a. Cations and anions in the polyelectrolyte migrate to positrode (positive electrode similar to cathode) and negatrode (negative electrode similar to anode) during the charge process and afterwards return to the previous state during the discharge step. Interestingly, the CV curves exhibit a 1.8 V potential window and, hence, all studies were achieved within the voltage window of 0 to 1.8 V at different scan rates (Fig. 6b) in which all curves show a couple of reversible redox peaks resulting from reversible redox-active reaction of active material. The current response improves with increasing current density, and CV curves are not significantly deviated from each other, attribute to a reduction in concentration polarization. The capacitance of the cell reaches 48.8 F g^−1^ at a scan rate of 2 mV s^−1^, indicating that cerium selenide structure can efficiently offer very many active channels in the solid-state electrolyte framework (Fig. 6c). Additionally, excess electrolyte migration sites are provided; the ion transport length is also shortened remarkably in the gel electrolyte system and, hence, the capacitance is much improved as the structure noticeably assists rapid ion transport.

The cyclic stability of the SC was studied by repeated CV cycles for 4000 times with a fixed large 100 mV s^−1^ scan rate as revealed in Fig. 6d. The designed cell exhibits a remarkable 85.5% cycling stability retention of original capacitance even at 4000 cycles, demonstrating the favorable reliability of our fabricated device. The cell shows a regular decrease in capacitance from the start to a couple of cycles with quite high reduction in capacitance due to the dehydration of residual water content in the polymer gel electrolyte. Therefore, the laboratory-scale cell demonstrates remarkable stability up to 4000 CV cycles. Nevertheless, the trivial instability of the cell can be simply controlled by improving the encapsulation on the cell to avoid the dehydration of the polymer gel electrolyte from electrode surfaces.

**Fig. 7:**
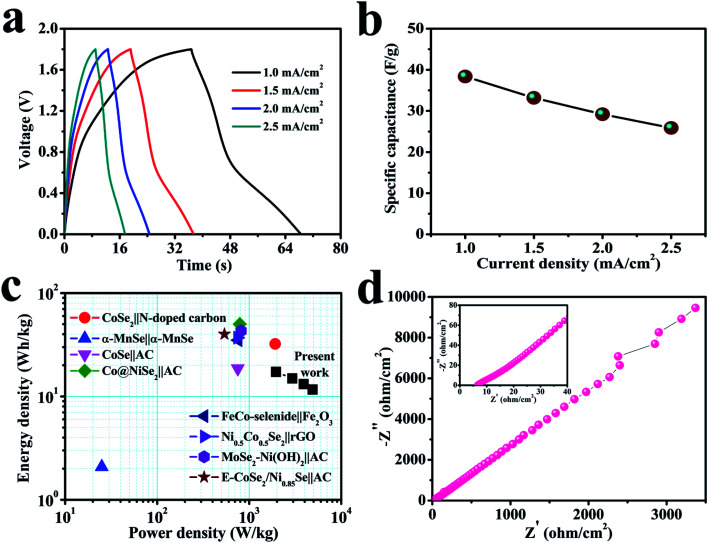
(a) GCD curves of the symmetric solid-state device at different current densities ranging from 1 to 2.5 mA cm^−2^. (b) Specific capacitance as a function of current density. (c) A Ragone plot. (d) A Nyquist plot from 100 kHz to 100 mHz. The inset shows a magnified view of the high-frequency region.

Fig. 7a shows the GCD curves of the cell at relatively high current densities (1–2.5 mA cm^−2^). The GCD plots with PVA–LiClO_4_ electrolyte exhibit a clear voltage drop, which may be because of the reasonably lower conductivity of the gel electrolyte.^69,70^ Non-triangular shape profile is obtained for the solid-state device, indicating a pseudo-capacitive behavior. Fig. 7b displays the dependence of capacitance for the device on the charge–discharge current density, which shows its capacitance changes from 38.4 to 25.9 F g^−1^ when the current density increases from 1 to 2.5 mA cm^−2^. The evaluated standards of capacitance allow the evaluation of power and energy densities, as they remain the crucial parameters in SC study. The power and energy densities of the fabricated SC cell are revealed in Fig. 7c, which displays superior energy and power output: when the power density increases from 1.96 to 4.89 kW kg^−1^, related energy density of the device changes from 17.26 to 11.63 W h kg^−1^. The power and energy densities for the fabricated SC have been calculated by considering the active mass loadings of both electrodes. According to the best of our knowledge, the cerium selenide‖cerium selenide SSC provides high power density compared to recently reported selenide-based symmetric and asymmetric SCs, such as CoSe_2_‖N-doped carbon nanowall ASC (1914.7 W kg^−1^, 2017),^71^ α-MnSe‖α-MnSe SSC (25 W kg^−1^, 2018),^72^ CoSe‖AC ASC (750 W kg^−1^, 2018),^15^ Co@NiSe_2_‖AC ASC (790 W kg^−1^, 2018),^73^ FeCo-selenide‖Fe_2_O_3_ ASC (759.6 W kg^−1^, 2018),^74^ Ni_0.5_Co_0.5_Se_2_‖rGO based ASC (745 W kg^−1^, 2018),^75^ MoSe_2_–Ni(OH)_2_‖AC based ASC (817 W kg^−1^, 2018),^76^ and E-CoSe_2_/Ni_0.85_Se‖AC ASC (538 W kg^−1^, 2018).^77^

**Fig. 8:**
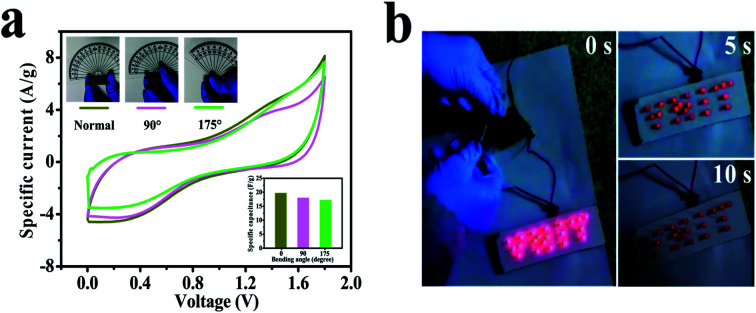
(a) CV curves of the symmetric solid-state device at various bending angles at a scan rate of 100 mV s^−1^. The inset shows the capacitance at the associated bending angles. (b) An LED panel powered by the manufactured cell for 10 s showing high intensity.

Furthermore, still low *R*_S_ (6.6 Ω) and *R*_CT_ are found in the high-frequency region of the Nyquist plot (Fig. 7d), signifying conductive electrode/electrolyte interface formation in spite of constructing a tandem solid-state system in which many extra resistive components have been included during cell fabrication.^78^ As a bendable energy storage device, it is capable of suffering various deformation modes, such as twisting, folding, or bending at diverse angles, without significant change in electrochemical activities.^79–81^Fig. 8a displays the CV curves at a scan rate of 100 mV s^−1^ for the bent SSC device even up to an angle of 175°. The change in capacitance is from 19.8 to 17.2 F g^−1^, maintaining 87% capacitive retention compared to normal state (inset, Fig. 8a). There are no noticeable deviations in CV shapes at various bending angles, suggesting exceptional mechanical flexibility and robustness of the fabricated cell. Also, the real-world application of the SC device was studied by charging for 30 s, and then effectively powering the logo of “VNIT” constructed with 21 LEDs (ESI video†). The LED panel emits a very bright light at the start of discharge and even glowed after 10 s (Fig. 8b), representing good power performance of the manufactured planar SC.

## 4. Conclusions

A cost-effective and simple chemical approach has been effectively utilized to synthesize cerium selenide in thin-film form, consisting of nano-pebbles embedded with nanoparticles, and capacitance of 285 F g^−1^ at 2 mA cm^−2^ in a liquid-state configuration with a potential limit of 0.9 V has been achieved. A flexible solid-state device was developed using symmetric cerium selenide electrodes sandwiched by polymer PVA–LiClO_4_ gel electrolyte to reduce the weight (and also the thickness) and widen the voltage window to 1.8 V with a higher power density of 4.89 kW kg^−1^ and capacity retention of 85.5% after 4000 CV cycles. The obtained results clearly demonstrate the practical applicability of cerium selenide electrodes for developing promising flexible SC devices for use in portable circuits/devices, such as the flashlights of smartphones, GPS trackers, and medical devices.

## Conflicts of interest

There are no conflicts to declare.

## Supplementary Material

NA-003-D0NA00893A-s001

NA-003-D0NA00893A-s002
