# Supplementary material for: The electrochemical kinetics of cerium selenide nano-pebbles: the design of a device-grade symmetric configured wide-potential flexible solid-state supercapacitor
Source: Nanoscale Adv. 2020 Dec 21;3(4):1057–66. doi: 10.1039/d0na00893a (PMC9417937; doi:10.1039/d0na00893a)
Supplement: NA-003-D0NA00893A-s001 [file NA-003-D0NA00893A-s001.pdf]

Electronic Supplementary Material (ESI) for Nanoscale Advances.
This journal is © The Royal Society of Chemistry 2020

Supplementary information

Electrochemical kinetics of cerium selenide nano-pebbles: Design of device grade symmetric configured wide-potential flexible solid-state supercapacitor

Bidhan Pandit,[ab] Akanksha Agarwal,[a] Priyanka Patel,[a] Babasaheb R. Sankapal*[a]

[a]Nano Materials and Device Laboratory, Department of Physics, Visvesvaraya National Institute of Technology, South Ambazari Road, Nagpur 440010, Maharashtra, India.

[b]Institut Charles Gerhardt Montpellier (ICGM), Université de Montpellier, CNRS, Place Eugène Bataillon, Montpellier, 34095, Cedex 5, France.

Corresponding author

Babasaheb R. Sankapal

E-mail: brsankapal@phy.vnit.ac.in; brsankapal@gmail.com

Tel.: +91 (712) 2801170; Fax No.:- +91 (712) 2223230

S1. Adhesion test

The adherence of the film was checked by using tixo-tape peel-off method. Initially, tixo-tape was stick on upper surface of film and then it was peel-off. The adherence of the film to the stainless steel (SS) substrate was so good that there was very minute change compare to original due to peel-off of some un-adhesive powdery particles from top-surface only. Photos show before and after peel-off image of the film on the SS substrate with no apparent change, exhibiting good adhesion.

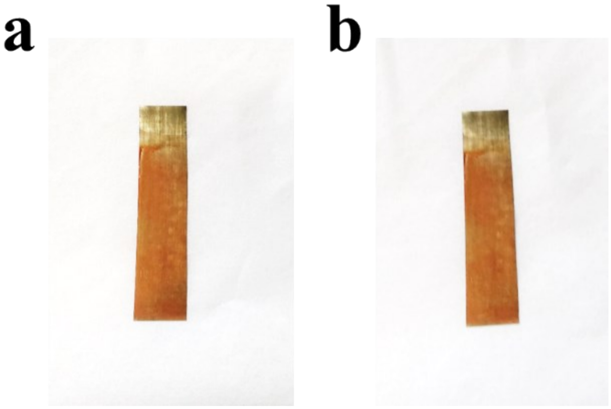

Figure S1 Electrode (a) before and (b) after peel-off test for adhesion.

S2. Contact angle analysis

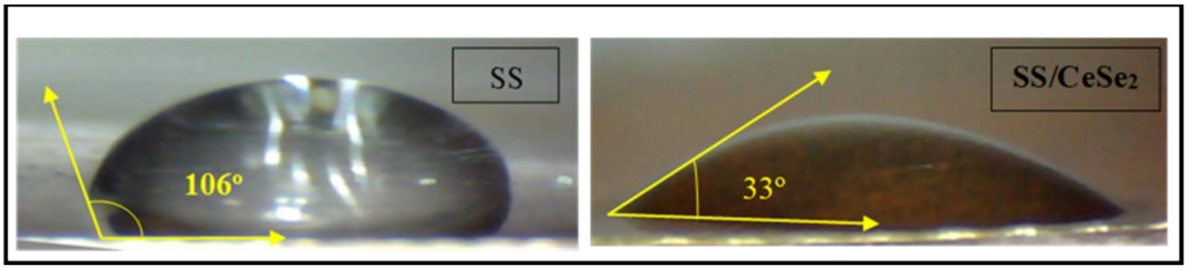

Figure S2: Contact angle for bare stainless steel (SS) substrate and SS/$CeSe_2$ film.

S3. Electrochemical characterizations

Specific capacitance[1] from the CV curves was calculated with the help of following formula:

$$C_s = \frac{1}{2mv\Delta V}\int_{V_i}^{V_f} I(V)dV \qquad (1)$$

where, 'C_s' signifies the specific capacitance (F/g), 'm' indicates mass (g) deposited on SS substrate, 'v' specifies scan rate (V), 'ΔV' is an functional potential frame and '$\int_{V_i}^{V_f} I(V)dV$' represents area under the CV curve.

Specific capacitance (C_s), specific energy (E) in Wh/kg and specific power (P) in W/kg from Galvanostatic charge-discharge can be calculated using following equations:

$$C_s = \frac{I\Delta t}{m\Delta V} \qquad (2)$$

$$E = \frac{1}{2}\left[\frac{C_s \Delta V^2}{3.6}\right] \qquad (3)$$

$$P = \frac{3600 \times E}{\Delta t} \qquad (4)$$

where, 'I' represents current intensity and 'Δt' denotes discharge time (t) of the experimental charge-discharge curve.

Table S1 Equivalent circuit parameters obtained from calculated EIS spectra for the electrode.

Material	R (Ω)	CPE		R (Ω)	W (Ω)	CPE		R (Ω)	Chi square
		Y_0 (F)	n			Y_0 (F)	n		
Cerium selenide	3.1	2.95×10^{-4}	0.86	9.24	3.91	17.15×10^{-4}	0.59	47.54	9.71×10^{-4}

References

1. S. Vijayakumar, S.-H. Lee and K.-S. Ryu, *Electrochim. Acta*, 2015, **182**, 979-986.
